# Goal Orientation and Emotional and Personality-Related Career Decision-Making Difficulties: The Mediating Role of Career Adaptability Among Chinese University Students

**DOI:** 10.3390/bs16050714

**Published:** 2026-05-06

**Authors:** Xiao Wang, Naiyi Wang, Jiaqing Wu, Guangqiang Wang

**Affiliations:** 1 Center for Teacher Education Research, Beijing Normal University, Beijing 100875, China; xiaowang@mail.bnu.edu.cn; 2 Faculty of Education, Beijing Normal University, Beijing 100875, China; 3 Department of Educational Management, Faculty of Education, East China Normal University, Shanghai 200062, China; bnu1010165@163.com

**Keywords:** goal orientation, career adaptability, emotional and personality-related career decision-making difficulties, the career construction model of adaptation, Chinese university students

## Abstract

Goal orientation is a key motivational factor in individuals’ career development, yet its links with emotional and personality-related career decision-making difficulties (EPCD) have received limited attention. According to the Career Construction Model of Adaptation (CCMA), this study examined how goal orientation relates to EPCD and whether four dimensions of career adaptability (CA)—career concern, career curiosity, career control, and career confidence—mediate these relationships. In total, 450 Chinese university students completed an online questionnaire measuring learning goal orientation (LGO), performance-approach (PAPG) and performance-avoidance goal orientations (PAVG), CA, and EPCD. The results showed that PAPG and PAVG were positively associated with EPCD, whereas LGO was not directly associated with EPCD. Mediation analyses indicated that concern, curiosity, and confidence mediated the relationships between LGO, PAPG, and EPCD; confidence mediated the relationship between PAVG and EPCD. The findings extend the application of CCMA and offer implications for career counseling and intervention for university students.

## 1. Introduction

Choosing a career is a central developmental task for university students. According to Super’s life-span theory (Super, 1980), university students are typically in the career exploration stage, during which they actively explore career paths and begin to form initial vocational choices. However, many students experience persistent indecision when faced with career choices. Compared with information-related difficulties, emotional and personality-related career decision-making difficulties (EPCD; Saka & Gati, 2007; Saka et al., 2008) represent a more enduring and severe form of career indecision, characterized by chronic anxiety, pessimistic views, as well as an unclear self-concept and identity. These difficulties are often linked to maladaptive outcomes, such as procrastination, low self-esteem, and depressive symptoms (Gadassi et al., 2015; Öztemel, 2014).

In China, EPCD may be particularly salient during the transition from university to work (Tian & Hou, 2023). Since the expansion of higher education in 1999, the number of university graduates has surged from 1.108 million in 2001 to 12.218 million in 2025 (National Bureau of Statistics of China, 2003, 2026). Meanwhile, China’s labor market is undergoing structural changes, including economic uncertainty, technological transformation, and rising unemployment (Zhang et al., 2025). In addition, collectivist cultural values and strong family expectations, partly shaped by the legacy of the one-child policy, may increase the psychological pressure associated with career decision-making (Zhu et al., 2023). Under these pressures, some students remain confident, while others experience persistent indecision and emotional difficulties, highlighting the need to better understand the psychological mechanisms underlying EPCD.

The Career Construction Model of Adaptation (CCMA; Savickas & Porfeli, 2012) provides a framework for understanding this process. The CCMA conceptualizes career development as a process of adaptation, in which individuals adjust to changing career circumstances through the interplay of adaptivity, adaptability, and adapting responses (Savickas, 2013). Specifically, adaptivity reflects individuals’ willingness and readiness to deal with career tasks, such as flexibility and openness to change. This adaptivity contributes to adaptability, a cluster of psychosocial resources that enable people to handle career challenges effectively (Savickas, 2013). These resources then shape individuals’ adapting responses, which are reflected in their attitudes and behaviors when approaching career tasks and transitions. From this perspective, EPCD can be understood as a maladaptive adapting response (Hirschi et al., 2015; Johnston, 2018) that arises when individuals lack sufficient motivational readiness and resources to cope with career tasks.

Goal orientation refers to a relatively stable tendency that shapes how individuals approach achievement-related tasks, including career tasks (Tolentino et al., 2014). According to achievement goal theory, individuals differ in how they define competence and success, and these differences influence their self-regulatory strategies as well as their affective and behavioral outcomes (Dweck & Leggett, 1988; Elliot & Church, 1997). Three classic types are commonly distinguished: learning goal orientation (LGO), which focuses on competence development; performance-approach goal orientation (PAPG), which emphasizes demonstrating competence; and performance-avoidance goal orientation (PAVG), which is driven by concerns about appearing incompetent (Elliot & Church, 1997; L. Wang & Yan, 2018). Consistent with prior career research (e.g., Johnston, 2018; Rudolph et al., 2017), goal orientation can be positioned as a motivational disposition that functions as an indicator of adaptivity within the CCMA framework. These different goal orientations reflect different forms of motivational readiness for career tasks, which may shape the development of adaptability resources and, in turn, influence adapting responses, such as EPCD (Johnston, 2018; Rudolph et al., 2017). Prior research has linked goal orientation to career indecision (P. A. Creed et al., 2009; Zhou & Xu, 2014), yet its associations with EPCD, an emotional and personality-related aspect of career indecision, remain underexplored.

Career adaptability (CA) refers to psychosocial resources that help individuals to navigate both present and anticipated career tasks and transitions (Savickas, 2013). Within the CCMA, CA helps to explain how motivational tendencies, such as goal orientation, are reflected in individuals’ responses to career-related challenges (Tolentino et al., 2014; Rudolph et al., 2017). Previous research indicates that different goal orientations are associated with varying levels of CA (Jeong & Hong, 2023), and CA is closely related to EPCD (D. Wang et al., 2020). However, it is still unclear whether CA plays a role in connecting goal orientation with EPCD. In addition, most studies examined CA as a global construct, with little attention to whether its four dimensions (concern, control, curiosity, and confidence) play different roles in connecting goal orientations to EPCD.

Despite growing research on career decision-making, three important gaps remain. First, although goal orientation has been linked to career-related outcomes, its association with EPCD remains insufficiently examined. Second, while the CCMA provides a useful framework linking adaptivity, adaptability resources, and adapting responses, little is known about whether goal orientation is linked to EPCD through career adaptability. Third, although career adaptability is multidimensional, existing research has given limited attention to whether its four dimensions play distinct roles in linking goal orientation to EPCD. To address these gaps, this study aims to examine how goal orientations relate to EPCD and whether four CA components mediate these relationships among Chinese university students.

### 1.1. Goal Orientation and EPCD

From the perspective of the CCMA, goal orientation reflects individuals’ motivational readiness for engaging in career-related tasks, corresponding to the adaptivity component of the model (Johnston, 2018). As different goal orientations involve distinct ways of interpreting challenges and regulating behavior, they are expected to show differential associations with career-related outcomes. In the present study, EPCD is conceptualized as a maladaptive adapting response, characterized by difficulties, anxiety, and indecision in the career decision-making process (Hirschi et al., 2015; Johnston, 2018). Accordingly, goal orientation may be directly related to EPCD.

Learning goal-oriented individuals usually perceive challenges as chances for learning and adopt more constructive coping strategies (Payne et al., 2007; Stasielowicz, 2019). In career-related situations, previous studies have linked LGO to stronger vocational identity (P. A. Creed & Hennessy, 2016), higher career decision-making self-efficacy (Garcia et al., 2012; Y. Jiang, 2021), and lower levels of depression, anxiety, and career indecision (P. A. Creed et al., 2009; Diaconu-Gherasim et al., 2024; Zhou & Xu, 2014). These findings suggest that LGO is an adaptive motivational tendency, with fewer negative attitudes and a clearer self-identity in career decision-making, which may serve as a buffer against these emotional difficulties.

PAPG directs attention towards demonstrating competence and seeking positive evaluation (Elliot & McGregor, 2001). Although PAPG can foster short-term effort to prove abilities, it may undermine sustained self-regulation when individuals face complex and uncertain career choices (Yousefi et al., 2011). A meta-analysis by Payne et al. (2007) reported that PAPG was more strongly related to better performance on simple and well-structured tasks than on complex and uncertain tasks. Under conditions of uncertainty, students high in PAPG may experience more anxiety and self-doubt, which are closely related to EPCD. Consistent with this reasoning, previous research has linked PAPG to higher levels of career indecision (P. A. Creed et al., 2009).

PAVA, driven by fear of failure and self-protection, reflects a defensive motivational pattern (Elliot & Church, 1997). PAVG has been consistently associated with maladaptive career outcomes, including weaker vocational identity (P. A. Creed & Hennessy, 2016), lower self-efficacy in career decision-making (P. Creed et al., 2013), and higher career indecision (P. A. Creed et al., 2009; Zhou & Xu, 2014). Individuals with this orientation are more likely to experience heightened anxiety, negative self-evaluations, and an unclear sense of self, which are core features of EPCD.

Overall, prior findings suggest that LGO is likely to be associated with lower levels of EPCD, whereas performance goal orientations, particularly PAVG, are likely to be associated with higher levels of EPCD.

**Hypothesis** **1.** *EPCD will be negatively associated with LGO.*

**Hypothesis** **2.** *EPCD will be positively associated with PAPG.*

**Hypothesis** **3.** *EPCD will be positively associated with PAVG.*

### 1.2. Goal Orientation, CA and EPCD

CA is defined as a set of psychosocial resources that enable individuals to effectively cope with career-related tasks and transitions, comprising concern, control, curiosity and confidence (Hirschi et al., 2015; Savickas & Porfeli, 2012). Within the CCMA, CA corresponds to the adaptability component, representing the resources individuals draw upon to regulate their responses to career challenges. Accordingly, CA is expected to be negatively associated with EPCD.

These four resources may play distinct roles in career decision-making (Savickas & Porfeli, 2012; Hirschi et al., 2015). Concern reflects future orientation and preparation, which may help reduce uncertainty about career choices. Control refers to a sense of agency and self-discipline, supporting an active and responsible approach to career tasks. Curiosity involves exploring possible vocational options, facilitating information-seeking about different alternatives. Confidence represents beliefs in one’s ability to solve problems and achieve career goals, which may alleviate anxiety and self-doubt during decision-making. Examining these dimensions separately may provide a more nuanced understanding of how CA relates to EPCD.

Prior research has consistently shown that CA is associated with fewer career decision-making difficulties. Hirschi et al. (2015) reported that all four CA dimensions were negatively associated with career decision difficulties among university students in Germany. Similarly, Jia et al. (2022) found that adaptability resources were linked to reduced anxiety and fewer problems in career decision-making. Importantly, D. Wang et al. (2020) observed negative associations between the four CA dimensions and EPCD among Chinese undergraduates. These findings suggest that higher levels of adaptability resources are associated with lower levels of EPCD.

**Hypothesis** **4.** *EPCD will be negatively associated with four CA dimensions.*

In addition, goal orientation provides a motivational foundation for the development of career adaptability. Previous research has primarily examined the link between goal orientation and the overall level of CA. The results consistently suggest that LGO is positively associated with CA, as individuals with LGO are more likely to engage actively in career exploration, exert self-control, and develop stronger confidence in career-related tasks (Tolentino et al., 2014; Son, 2018). PAPG has also been linked to higher levels of CA (Yousefi et al., 2011), as individuals with PAPG tend to invest more effort in career-related activities to demonstrate competence, such as pursuing higher aspirations (P. Creed et al., 2011), engaging in active career exploration (P. Creed et al., 2013), and adopting career-enhancing strategies (P. A. Creed & Hennessy, 2016). In contrast, PAVG reflects concerns about making mistakes and avoiding negative evaluations, and is generally associated with lower levels of adaptability (Yousefi et al., 2011; Jeong & Hong, 2023).

**Hypothesis** **5.** *LGO will be positively associated with the four dimensions of CA.*

**Hypothesis** **6.** *PAPG will be positively associated with the four dimensions of CA.*

**Hypothesis** **7.** *PAVG will be negatively associated with the four dimensions of CA.*

Building on the CCMA (Savickas & Porfeli, 2012) and the evidence reviewed above, CA may serve as a key mechanism linking goal orientation to EPCD. Specifically, goal orientation, as a form of motivational readiness, may influence the development of adaptability resources, which in turn shape individuals’ career-related responses. Accordingly, the four dimensions of career adaptability are expected to mediate the associations among LGO, PAPG, PAVG, and EPCD. More specifically, LGO and PAPG may be associated with lower levels of EPCD through stronger adaptability resources, whereas PAVG may be associated with higher levels of EPCD through weaker adaptability resources.

**Hypothesis** **8.** *The four dimensions of CA will mediate the association between LGO and EPCD.*

**Hypothesis** **9.** *The four dimensions of CA will mediate the association between PAPG and EPCD.*

**Hypothesis** **10.** *The four dimensions of CA will mediate the association between PAVG and EPCD.*

## 2. Materials and Methods

### 2.1. Participants and Procedures

The survey was carried out during the winter vacation in January 2021. During this period, students were preparing for the upcoming spring recruitment. Participants were recruited from universities in Beijing, Tianjin, and Wuhan. These three regions have relatively developed higher education in China. Using convenience sampling, participants were invited through university WeChat groups (a primary social networking tool in China) and campus electronic bulletins with the support of trained research assistants. To encourage participation, participants were given a small token of appreciation, approximately 5–10 Chinese Yuan (RMB). Data were collected online through the Wenjuanxing platform, widely used for academic surveys in China. Before the questionnaire survey was carried out, each participant received a statement of informed consent. Participants were told about the right to discontinue anytime and the anonymity of all responses.

In total, 462 questionnaires were collected. Of these, 450 were retained after the exclusion of patterned or incomplete data. Participants were aged 18 to 28 (mean (*M*) = 22.59, standard deviation (*SD*) = 1.87). Among the recruited participants, 140 (31.11%) were male, and 310 (68.89%) were female. In terms of major, 234 (52.00%) participants studied humanities and social sciences, and 216 (48.00%) studied science and engineering. As regards grade, 196 (43.56%) participants were junior or senior undergraduates, 242 (53.78%) were postgraduates, and 12 (2.67%) did not report. The participants were largely in stages of career exploration and preparation for the school-to-work transition.

### 2.2. Measures

#### 2.2.1. Goal Orientation Scale

GO was assessed using the Chinese version of the Achievement Goals Questionnaire (J. C. Jiang, 2004), adapted from Elliot and Church (1997). The questionnaire included 16 items and three subscales: LGO (five items), PAPG (six items) and PAVG (five items). Example items were as follows: “I hope to learn as much as possible” (learning), “Doing better than other students is important to me” (performance-approach) and “I am often motivated by my fear of performing poorly” (performance-avoidance). Participants rated each item on a five-point scale, with higher scores reflecting a stronger tendency toward specific goal orientation. The Cronbach’s alpha coefficients were 0.74, 0.89 and 0.80 for LGO, PAPG and PAVG, respectively. The confirmatory factor analysis (CFA) indicated an acceptable construct validity: *χ*^2^/*df* = 2.68, CFI = 0.95, TLI = 0.93, and RMSEA = 0.06.

#### 2.2.2. Career Adaptability Scale

The assessment of CA was performed through the Career Adapt-Abilities Scale-Short Form (CAAS-SF China; Yu et al., 2020). The short form was adopted to reduce participant burden and improve response quality in an online survey context while still capturing the core dimensions of career adaptability. Previous studies have demonstrated that the CAAS-SF shows acceptable reliability and validity across different cultural contexts, including Chinese samples (Yu et al., 2020). CAAS-SF consisted of four three-item subscales: concern (e.g., “considering what my future will be like”), control (e.g., “depending on myself”), confidence (e.g., “paying attention to handling affairs well”) and curiosity (e.g., “observing multiple ways of handling affairs”). The responses were rated on a five-point scale, with higher scores indicating stronger CA. The total scale’s Cronbach’s alpha coefficient was 0.84, and subscales were 0.72 (concern), 0.68 (confidence), 0.57 (control), and 0.54 (curiosity). Since Cronbach’s alpha can be sensitive to the number of items, we further examined the mean inter-item correlations (MICs). The MICs ranged from 0.28 to 0.48, falling within the recommended range of 0.15–0.50 for brief scales (Clark & Watson, 1995). Additionally, CFA supported the four-factor structure, with acceptable model fit: *χ*^2^/*df* = 2.52, CFI = 0.95, TLI = 0.93, and RMSEA = 0.06.

#### 2.2.3. EPCD Scale

EPCD was assessed using the EPCD Scale-Short Form (EPCD-SF; Hou et al., 2016). The scale comprises 23 items (one warm-up and two validity-check items) and three subscales: pessimistic views (six items), self-concept and identity (six items), and anxiety (eight items). It was based on a nine-point Likert scale, with higher scores indicating stronger EPCD. This scale included items such as “Very few careers are truly interesting” (pessimistic views), “I am concerned about having to cope with the complicated process of making career decisions” (anxiety) and “I still have no idea of my career interests” (self-concept and identity). The total scale’s Cronbach’s alpha coefficient was 0.91, and the subscales were 0.72 to 0.88. The CFA indicated an acceptable construct validity: *χ*^2^/*df* = 3.25, CFI = 0.92, TLI = 0.91 and RMSEA = 0.07.

### 2.3. Data Analyses

SPSS 27.0 and AMOS 26.0 were used to perform all statistical analyses. First, the association between demographic variables and EPCD was tested. Then, the common method bias was examined through Harman’s single-factor test (Harman, 1976). Associations among study variables were explored using correlation analysis and descriptive statistics. In addition, structural equation modeling (SEM) was adopted to examine the hypothesized mediation model. Model fit was assessed based on the following indices: *χ*^2^/*df* < 3, CFI > 0.9, RMSEA < 0.08 and SRMR < 0.08 (Hu & Bentler, 1999; Schumacker & Lomax, 2004). Finally, indirect effects were examined via a bias-corrected bootstrapping procedure with 5000 resamples (Preacher & Hayes, 2008). The mediation was supported when the 95% confidence interval (CI) was exclusive of zero.

## 3. Results

### 3.1. Preliminary Analysis

Preliminary analysis showed that age was negatively associated with EPCD (*r* = −0.11, *p* < 0.05), with older students reporting slightly lower-level EPCD. Group comparisons showed no significant differences in EPCD across gender, major, and grade. Therefore, age was taken as a control variable in the final structural model. Harman’s single-factor test indicated no substantial common method bias, yielding 12 factors with eigenvalues above 1, of which the first accounted for 18.20% of the total variance.

### 3.2. Descriptive Statistics

Table 1 reports the descriptive statistics for all variables. EPCD was negatively related to LGO but positively related to PAPG and PAVG. Among the adaptability resources, career concern, control, and confidence showed small negative correlations with EPCD, whereas career curiosity was not significantly related to EPCD. Regarding correlations with goal orientations, all four adaptability dimensions were positively correlated with LGO and PAPG, but none was significantly related to PAVG. All variables were significantly correlated, except for the correlations between PAVG and the four CA subdimensions and between career curiosity and EPCD.

### 3.3. SEM Analysis Testing the Mediating Role of Four CA Dimensions

For the purpose of testing the hypothesized model, SEM was conducted with three goal orientations (LGO, PAPG, and PAVG) as independent variables, EPCD as the outcome, and four subdimensions of CA as observed mediators. Age was entered as a control variable. The model demonstrated acceptable fit to the data: *χ*^2^/*df* = 2.68, CFI = 0.99, TLI = 0.93, SRMR = 0.03 and RMSEA = 0.06.

**Figure 1:**
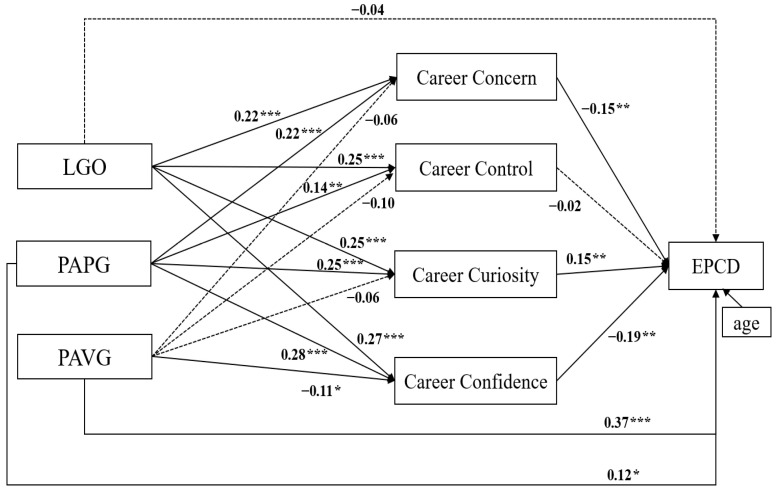
SEM of goal orientation, CA and EPCD. * *p* < 0.05, ** *p* < 0.01, *** *p* < 0.001. LGO = learning goal orientation; PAPG = performance-approach goal orientation; PAVG = performance-avoidance goal orientation; EPCD = emotional and personality-related career decision-making difficulties.

Figure 1 illustrates the structural model with standardized path coefficients. LGO was not significantly associated with EPCD (*β* = −0.04, *p* > 0.05); whereas PAPG and PAVG were associated with higher EPCD (*β* = 0.12, *p* < 0.05; *β* = 0.37, *p* < 0.001). H1 was not supported, H2 and H3 were supported. Regarding the mediators, career concern and career confidence were negatively associated with EPCD (*β* = −0.15, *p* < 0.01; *β* = −0.19, *p* < 0.01), while career curiosity was positively associated with EPCD (*β* = 0.15, *p* < 0.01). Career control was not significantly associated with EPCD (*β* = −0.02, *p* > 0.05). H4 was partially supported. In addition, LGO and PAPG were positively associated with four CA dimensions: career concern (*β* = 0.22, *p* < 0.001; *β* = 0.22, *p* < 0.001), career control (*β* = 0.25, *p* < 0.001; *β* = 0.14, *p* < 0.01), career curiosity (*β* = 0.25, *p* < 0.001; *β* = 0.25, *p* < 0.001), and career confidence (*β* = 0.27, *p* < 0.001; *β* = 0.28, *p* < 0.001). PAVG was negatively associated with career confidence (*β* = −0.11, *p* < 0.05). H5 and H6 were supported, whereas H7 was partially supported.

A bias-corrected bootstrapping procedure was applied to examine the mediation effect (Table 2). LGO was associated with EPCD via career concern, curiosity, and confidence (*β* = −0.03, 95% CI = [−0.07, −0.01], *β* = 0.04, 95% CI = [0.01, 0.08], *β* = −0.05, 95% CI = [−0.09, −0.02], respectively), which suggested full mediation given its nonsignificant direct path. PAPG was also significantly associated with EPCD through career concern, curiosity, and confidence (*β* = −0.03, 95% CI = [−0.07, −0.01], *β* = 0.04, 95% CI = [0.01, 0.08], *β* = −0.05, 95% CI = [−0.10, −0.02], respectively), which indicated partial mediation. PAVG was only significantly associated with EPCD through career confidence (*β* = 0.03, 95% CI = [0.01, 0.07]), indicating partial mediation. Together, the three goal orientations demonstrated distinct indirect pathways to EPCD through different CA dimensions. H8, H9 and H10 were partially supported.

## 4. Discussion

The present study extended the literature by examining how distinct achievement goal orientations (i.e., LGO, PAPG, PAVG) relate to EPCD and whether the four CA dimensions mediated these relationships. Overall, the findings showed that PAPG and PAVG were positively linked to EPCD, whereas LGO showed no direct effect on EPCD. In addition, LGO and PAPG were positively related to all four adaptability resources, whereas PAVG was related only to career confidence. Furthermore, concern and confidence were related to lower EPCD, curiosity was related to higher EPCD, whereas control showed no significant association with EPCD. Finally, concern, confidence, and curiosity mediated the links between LGO, PAPG and EPCD, whereas only confidence mediated the link between PAVG and EPCD.

Consistent with prior studies (P. A. Creed et al., 2009; Zhou & Xu, 2014), LGO showed a small negative correlation with EPCD. However, when adaptability resources were included in the model, LGO was no longer significantly associated with EPCD. This result suggests that its protective effect on EPCD operates primarily through the development of career concern and confidence. In contrast, both PAPG and PAVG were positively associated with EPCD, in line with earlier research linking performance goal orientations to career indecision (P. A. Creed et al., 2009). According to achievement goal theory (Elliot & McGregor, 2001), performance goal orientations are characterized by a shared evaluative focus, with competence defined in relation to external standards and others’ judgments. In the career domain, such evaluative concerns may increase the emotional burden of career decision-making, rendering it more self-threatening and anxiety-provoking (Burnette et al., 2013; Payne et al., 2007). These findings suggest that performance goal orientations are more directly associated with EPCD than LGO, which operates indirectly through adaptability resources.

The associations between goal orientations and the four adaptability resources showed that LGO and PAPG were each positively linked to all four adaptability dimensions, consistent with prior studies (Jeong & Hong, 2023; Tolentino et al., 2014; Yousefi et al., 2011). Although LGO and PAPG differ in their underlying motivations, both may sustain active engagement in career tasks. Students with LGO tend to view career development as an ongoing process of self-improvement, which may foster future planning, exploration, responsibility, and confidence in dealing with career-related tasks. Students high in PAPG also invest in career preparation, although their engagement is driven by the desire to demonstrate competence and outperform peers. By contrast, PAVG was linked only to lower career confidence, suggesting that concerns about failure and negative evaluation may undermine students’ beliefs in their ability to make career decisions (Jeong & Hong, 2023). This pattern indicates that approach-oriented goals support adaptability resources, whereas avoidance-oriented goals may weaken confidence.

Consistent with the CCMA (Savickas & Porfeli, 2012) and prior studies (Jia et al., 2022; D. Wang et al., 2020), career concern and career confidence were negatively associated with EPCD. Career concern refers to paying attention to one’s future and making plans ahead; career confidence denotes the conviction that one can effectively solve problems and complete career tasks (Savickas & Porfeli, 2012). Students who maintain a clear future direction and strong confidence tend to sustain optimism and self-efficacy in an uncertain career environment (Hirschi et al., 2015; Chui et al., 2022; Parola et al., 2025). Consequently, they are less likely to experience pessimism, anxiety, and self-doubt in making career choices.

Career curiosity was positively related to EPCD, in contrast to prior studies reporting negative or nonsignificant associations (Jia et al., 2022; Karacan-Ozdemir, 2019; D. Wang et al., 2020). This unexpected result suggests that career exploration may not always function as a protective resource. Instead, it may expose individuals to a wider range of options, conflicting information, and increased decisional ambiguity, thereby amplifying rather than resolving emotional tension (Storme & Celik, 2018; Vignoli, 2015; Xu & Tracey, 2014). This finding should be interpreted with caution and may reflect the specific context of the present study rather than a general pattern. In China’s highly competitive employment context, greater curiosity may sometimes intensify these difficulties rather than alleviate them. Career control, by contrast, showed no significant association with EPCD, although it has generally been regarded as an important adaptability resource (Hirschi et al., 2015; Jia et al., 2022). One possible explanation is that control reflects agentic self-regulation and a sense of personal responsibility over one’s career, whereas agency alone may be insufficient to address EPCD, which is characterized by chronic anxiety, pessimistic appraisals, and identity-related uncertainty (Gati et al., 2011). This pattern may also be contextually shaped, as external pressures and limited opportunities may weaken the role of personal agency in reducing EPCD. Collectively, these findings suggest that the four dimensions of career adaptability may not function uniformly in relation to EPCD in the present Chinese sample. At the same time, the findings for career curiosity and career control may reflect both contextual influences and possible measurement limitations related to the relatively low internal consistency of these two subscales.

Taken together, the findings suggest that distinct goal orientations are linked to EPCD through different adaptability resources. LGO was associated with lower EPCD through stronger concern and confidence, whereas PAVG was linked to higher EPCD through lower career confidence. A notable finding is that PAPG displayed a more complex motivational profile. Its positive direct association with EPCD suggests that an evaluative focus on outperforming others may heighten vulnerability to emotional career difficulties, while its positive associations with all four adaptability resources indicate that PAPG simultaneously mobilizes active career preparation and resource development. In this sense, PAPG should not be viewed as purely adaptive or maladaptive in the career domain but as a motivational tendency whose adaptive value depends on the balance between resource-building and evaluative pressure.

Finally, these findings should be interpreted within the specific sociocultural context of China. Chinese university students often face intense academic pressure, strong parental expectations, and a highly competitive labor market (Leung et al., 2011; Ni et al., 2024). In such a context, performance goal orientations may be reinforced by external evaluation systems, while the high stakes attached to educational and occupational choices may amplify the emotional costs of career uncertainty. This may help explain why performance goal orientations were more directly related to EPCD, why curiosity did not appear to be protective, and why control was not significantly associated with EPCD in the present study. Future research could further examine how these contextual factors interact with individual motivational and adaptability resources in shaping EPCD.

### 4.1. Theoretical Implications

The present study has three main theoretical implications for research on career indecision. First, by focusing specifically on EPCD, this study extends understanding of career indecision beyond informational and cognitive difficulties. This is theoretically important because it highlights that career indecision is not only a matter of insufficient information or ineffective decision strategies but may also involve deeper emotional and personality-related processes in the career decision-making process. Second, this study contributes by conceptualizing EPCD from a motivational perspective. Previous research has mainly explained EPCD in terms of personality traits, negative emotions, or general decision-making problems (Gati et al., 2011). By integrating achievement goal theory into the CCMA, goal orientation is positioned as an important adaptivity-related disposition for understanding EPCD. In particular, the different associations of LGO, PAPG, and PAVG with EPCD further suggest that distinct forms of motivation may be linked to EPCD in different ways.

Third, this study contributes to the CCMA literature by examining the mediating roles of the four dimensions of CA in the associations between goal orientations and EPCD. Rather than conceptualizing career adaptability as a single global resource, this study shows that its dimensions may play differentiated roles in linking motivational dispositions to EPCD. Specifically, concern and confidence were associated with lower EPCD, whereas curiosity showed a more complex positive association, and control was not significantly related to EPCD. These results suggest that the four adaptability dimensions may represent distinct self-regulatory resources in the process through which goal orientations relate to EPCD. This extends the CCMA by highlighting the differentiated functions of adaptability components in explaining emotional and identity-related career difficulties.

### 4.2. Practical Implications

The present findings offer several practical implications for career education and counseling in Chinese universities, where students often make career decisions under strong labor market pressure and parental expectations.

First, universities may need to pay closer attention to students’ goal orientations in career education and counseling. Although career courses in many Chinese universities already include self-exploration and career planning, they often pay less attention to the motivational patterns that shape how students approach career choice. The present findings suggest that students may benefit from being encouraged to view career choice as a developmental process involving self-understanding and person–environment fit, rather than as a high-stakes judgment of personal competence and success. This perspective can be made more explicit in career courses and employment guidance. For students with stronger PAPG, counselors might help reduce excessive reliance on external prestige and comparison while placing greater emphasis on personal growth and competence development. For students with stronger PAVG, more support may be needed to address fear of failure, excessive self-judgment, and the pressure associated with making the “right” choice. Such support may be provided through career courses, as well as group or individual career counseling.

Second, interventions may need to target specific adaptability resources. Given the negative associations of career concern and confidence with EPCD, it may be beneficial to provide career support earlier in the undergraduate years rather than concentrating it in the final year, when decision pressure is often greatest. Activities such as lifeline mapping, scenario planning, and alumni mentoring may help students develop a clearer sense of direction and strengthen their confidence in handling career-related challenges. At the same time, the positive association between career curiosity and EPCD suggests that exploration by itself may not always be helpful. In China’s rapidly changing labor market, more information does not always bring greater clarity. Therefore, exploratory activities should be accompanied by opportunities for reflection, so that students can connect what they learn from exploration with their own values, priorities, and long-term goals. In this way, career exploration may become more helpful in clarifying choices rather than adding further uncertainty.

### 4.3. Limitations and Future Directions

Several limitations should be noted. First, because the data were cross-sectional, the relationships among goal orientations, CA, and EPCD cannot be interpreted causally. In addition, all variables were measured using self-report questionnaires. Although common method bias did not appear to be a serious concern, future research could adopt longitudinal designs and multiple data sources to test these relationships more rigorously.

Second, participants were recruited through convenience sampling, which may have introduced sampling bias and limited the generalizability of the findings. Because the sample was drawn from universities in major cities, the results may not fully generalize to students from less-developed regions. Future studies could use more diverse samples to examine the generalizability of the findings across different regional and institutional contexts.

Third, the sample included only Chinese university students. Given the highly competitive career context in China, caution is needed when generalizing the findings to other cultural or educational settings. Future Research in different sociocultural contexts would help determine whether similar patterns emerge under varying competitive conditions and meanings attached to career choice.

Finally, the findings related to career control and career curiosity should be interpreted with caution, given the relatively low internal consistency of these two subscales. Future studies could use the full 24-item CAAS (Hou et al., 2012) to improve measurement precision and further examine the robustness of these findings. Overall, more refined measurement approaches may help clarify the roles of different adaptability dimensions in relation to EPCD.

## 5. Conclusions

This study investigated how different goal orientations are related to EPCD and whether CA helps explain this relationship among Chinese university students. The findings indicate that learning, performance-approach, and performance-avoidance goal orientations are associated with EPCD in different ways. LGO was linked to lower EPCD via career concern and confidence. PAPG showed a mixed pattern: it was associated with EPCD while also supporting career concern and confidence that were linked to lower EPCD. PAVG was directly associated with higher EPCD and was also partly linked to EPCD through reduced career confidence. Overall, these findings highlight the differentiated roles of goal orientations and adaptability resources in understanding EPCD.

## Figures and Tables

**Table 1:** Descriptive statistics among goal orientations, CA dimensions, and EPCD.

Variables	*M*	*SD*	1	2	3	4	5	6	7	8
LGO	3.57	0.65	1							
PAPG	3.52	0.86	0.14 **	1						
PAVG	3.29	0.88	−0.12 **	0.48 ***	1					
Career Concern	3.87	0.68	0.26 ***	0.22 ***	0.02	1				
Career Control	4.06	0.54	0.29 ***	0.13 **	−0.07	0.48 ***	1			
Career Curiosity	3.99	0.54	0.29 ***	0.26 ***	0.03	0.49 ***	0.42 ***	1		
Career Confidence	3.94	0.55	0.33 ***	0.27 ***	−0.01	0.54 ***	0.49 ***	0.60 ***	1	
EPCD	5.80	1.21	−0.14 **	0.25 ***	0.45 ***	−0.17 ***	−0.15 **	−0.01	−0.17 ***	1

** *p* < 0.01; *** *p* < 0.001. *M* = mean; *SD* = standard deviation; LGO = learning goal orientation; PAPG = performance-approach goal orientation; PAVG = performance-avoidance goal orientation; EPCD = emotional and personality-related career decision-making difficulties.

**Table 2:** Path estimate (*SE*) and bootstrapped 95% CIs for the mediation model.

Path	Effect	*SE*	*p*	Lower 95% CI	Upper 95% CI
Direct effect	data				
LGO-EPCD	−0.042	0.083	0.393	−0.131	0.048
PAPG-EPCD	0.1200	0.07	0.026	0.015	0.219
PAVG-EPCD	0.375	0.07	0.001	0.266	0.472
Indirect effect					
LGO-career concern-EPCD	−0.033	0.014	0.002	−0.067	−0.012
LGO-career control-EPCD	−0.006	0.013	0.608	−0.033	0.018
LGO-career curiosity-EPCD	0.039	0.017	0.005	0.011	0.079
LGO-career confidence-EPCD	−0.051	0.018	0.001	−0.092	−0.020
PAPG-career concern-EPCD	−0.034	0.015	0.002	−0.070	−0.012
PAPG-career control-EPCD	−0.003	0.007	0.534	−0.022	0.009
PAPG-career curiosity-EPCD	0.038	0.017	0.004	0.011	0.077
PAPG-career confidence-EPCD	−0.052	0.019	0.001	−0.095	−0.021
PAVG-career concern-EPCD	0.009	0.008	0.146	−0.004	0.029
PAVG-career control-EPCD	0.002	0.006	0.477	−0.006	0.018
PAVG-career curiosity-EPCD	−0.009	0.009	0.186	−0.034	0.005
PAVG-career confidence-EPCD	0.020	0.011	0.016	0.004	0.048

*N* = 450. CI = confidence interval; *SE* = standard error.

## Data Availability

Data will be made available on request (the data are not publicly available due to privacy).

## Abbreviations

The following abbreviations are used in this manuscript:

EPCD	Emotional and personality-related career decision-making difficulties
CA	Career Adaptability
LGO	Learning goal orientation
PAPG	Performance-approach goal orientation
PAVG	Performance-avoidance goal orientation

## References

[B1-behavsci-16-00714] Burnette J. L., O’Boyle E. H., VanEpps E. M., Pollack J. M., Finkel E. J. (2013). Mind-sets matter: A meta-analytic review of implicit theories and self-regulation. Psychological Bulletin.

[B2-behavsci-16-00714] Chui H., Li H., Ngo H. (2022). Linking protean career orientation with career optimism: Career adaptability and career decision self-efficacy as mediators. Journal of Career Development.

[B3-behavsci-16-00714] Clark L. A., Watson D. (1995). Constructing validity: Basic issues in objective scale development. Psychological Assessment.

[B4-behavsci-16-00714] Creed P., Buys N., Tilbury C., Crawford M. (2013). The relationship between goal orientation and career striving in young adolescents. Journal of Applied Social Psychology.

[B5-behavsci-16-00714] Creed P., Tilbury C., Buys N., Crawford M. (2011). Cross-lagged relationships between career aspirations and goal orientation in early adolescents. Journal of Vocational Behavior.

[B6-behavsci-16-00714] Creed P. A., Fallon T., Hood M. (2009). The relationship between career adaptability, person and situation variables, and career concerns in young adults. Journal of Vocational Behavior.

[B7-behavsci-16-00714] Creed P. A., Hennessy D. A. (2016). Evaluation of a goal orientation model of vocational identity. The Career Development Quarterly.

[B8-behavsci-16-00714] Diaconu-Gherasim L. R., Elliot A. J., Zancu A. S., Brumariu L. E., Măirean C., Opariuc-Dan C., Crumpei-Tanasă I. (2024). A meta-analysis of the relations between achievement goals and internalizing problems. Educational Psychology Review.

[B9-behavsci-16-00714] Dweck C. S., Leggett E. L. (1988). A social-cognitive approach to motivation and personality. Psychological Review.

[B10-behavsci-16-00714] Elliot A. J., Church M. A. (1997). A hierarchical model of approach and avoidance achievement motivation. Journal of Personality and Social Psychology.

[B11-behavsci-16-00714] Elliot A. J., McGregor H. A. (2001). A 2 × 2 achievement goal framework. Journal of Personality and Social Psychology.

[B12-behavsci-16-00714] Gadassi R., Waser A., Gati I. (2015). Gender differences in the association of depression with career indecisiveness, career-decision status, and career-preference crystallization. Journal of Counseling Psychology.

[B13-behavsci-16-00714] Garcia P. R. J. M., Restubog S. L. D., Toledano L. S., Tolentino L. R., Rafferty A. E. (2012). Differential moderating effects of student- and parent-rated support in the relationship between learning goal orientation and career decision-making self-efficacy. Journal of Career Assessment.

[B14-behavsci-16-00714] Gati I., Gadassi R., Saka N., Hadadi Y., Ansenberg N., Friedmann R., Asulin-Peretz L. (2011). Emotional and personality-related aspects of career decision-making difficulties: Facets of career indecisiveness. Journal of Career Assessment.

[B15-behavsci-16-00714] Harman H. H. (1976). Modern factor analysis.

[B16-behavsci-16-00714] Hirschi A., Herrmann A., Keller A. C. (2015). Career adaptivity, adaptability, and adapting: A conceptual and empirical investigation. Journal of Vocational Behavior.

[B17-behavsci-16-00714] Hou Z. J., Leung S. A., Li X., Li X., Xu H. (2012). Career adapt-abilities scale—China form: Construction and initial validation. Journal of Vocational Behavior.

[B18-behavsci-16-00714] Hou Z. J., Li X., Liu Y.-L., Gati I. (2016). The emotional and personality-related career decision-making difficulties questionnaire—Validation of the Chinese version. Journal of Career Assessment.

[B19-behavsci-16-00714] Hu L., Bentler P. M. (1999). Cutoff criteria for fit indexes in covariance structure analysis: Conventional criteria versus new alternatives. Structural Equation Modeling.

[B20-behavsci-16-00714] Jeong C., Hong A. J. (2023). Career adaptability and mediated social network process linking achievement goal orientation to behavior. Journal of Employment Counseling.

[B21-behavsci-16-00714] Jia Y., Hou Z. J., Zhang H., Xiao Y. (2022). Future time perspective, career adaptability, anxiety, and career decision-making difficulty: Exploring mediations and moderations. Journal of Career Development.

[B22-behavsci-16-00714] Jiang J. C. (2004). Relations between achievement goal orientation and class motivation climate, learning strategy, academic performance. Unpublished master’s thesis.

[B23-behavsci-16-00714] Jiang Y. (2021). Learning goal orientation, career self-efficacy, and career interest: A moderated mediation model. Journal of Employment Counseling.

[B24-behavsci-16-00714] Johnston C. S. (2018). A systematic review of the career adaptability literature and future outlook. Journal of Career Assessment.

[B25-behavsci-16-00714] Karacan-Ozdemir N. (2019). Associations between career adaptability and career decision-making difficulties among Turkish high school students. International Journal for Educational and Vocational Guidance.

[B26-behavsci-16-00714] Leung S. A., Hou Z.-J., Gati I., Li X. (2011). Effects of parental expectations and cultural-values orientation on career decision-making difficulties of Chinese university students. Journal of Vocational Behavior.

[B27-behavsci-16-00714] National Bureau of Statistics of China (2003). Statistical communique of the People’s Republic of China on the 2002 national economic and social development.

[B28-behavsci-16-00714] National Bureau of Statistics of China (2026). Statistical communiqué of the People’s Republic of China on the 2025 national economic and social development.

[B29-behavsci-16-00714] Ni J., Liu A., Shi Y., Guo J. (2024). Impact of academic involution atmosphere on college students’ mental exhaustion: A chain mediation model. International Journal of Educational Research.

[B30-behavsci-16-00714] Öztemel K. (2014). Career indecisiveness of Turkish high school students: Associations with personality characteristics. Journal of Career Assessment.

[B31-behavsci-16-00714] Parola A., Pettignano M., Marcionetti J. (2025). The role of character strengths of wisdom, career adaptability and optimism on future orientation. International Journal for Educational and Vocational Guidance.

[B32-behavsci-16-00714] Payne S. C., Youngcourt S. S., Beaubien J. M. (2007). A meta-analytic examination of the goal orientation nomological net. Journal of Applied Psychology.

[B33-behavsci-16-00714] Preacher K. J., Hayes A. F. (2008). Asymptotic and resampling strategies for assessing and comparing indirect effects in multiple mediator models. Behavior Research Methods.

[B34-behavsci-16-00714] Rudolph C. W., Lavigne K. N., Zacher H. (2017). Career adaptability: A meta-analysis of relationships with measures of adaptivity, adapting responses, and adaptation results. Journal of Vocational Behavior.

[B35-behavsci-16-00714] Saka N., Gati I. (2007). Emotional and personality-related aspects of persistent career decision-making difficulties. Journal of Vocational Behavior.

[B36-behavsci-16-00714] Saka N., Gati I., Kelly K. R., Kelly K. R. (2008). Emotional and personality-related aspects of career decision-making difficulties. Journal of Career Assessment.

[B37-behavsci-16-00714] Savickas M. L., Brown S. D., Lent R. W. (2013). Career construction theory and practice. Career development and counseling: Putting theory and research into work.

[B38-behavsci-16-00714] Savickas M. L., Porfeli E. J. (2012). Career adapt-abilities scale: Construction, reliability, and measurement equivalence across 13 countries. Journal of Vocational Behavior.

[B39-behavsci-16-00714] Schumacker R. E., Lomax R. G. (2004). A beginner’s guide to structural equation modeling.

[B40-behavsci-16-00714] Son S. (2018). The more reflective, the more career-adaptable: A two-wave mediation and moderation analysis. Journal of Vocational Behavior.

[B41-behavsci-16-00714] Stasielowicz L. (2019). Goal orientation and performance adaptation: A meta-analysis. Journal of Research in Personality.

[B42-behavsci-16-00714] Storme M., Celik P. (2018). Career exploration and career decision-making difficulties: The moderating role of creative self-efficacy. Journal of Career Assessment.

[B43-behavsci-16-00714] Super D. E. (1980). A life-span, life-space approach to career development. Journal of Vocational Behavior.

[B44-behavsci-16-00714] Tian L., Hou Z. (2023). Gender discrimination and career decision-making difficulties among female Chinese college students: The buffering role of coping styles. Career Development Quarterly.

[B45-behavsci-16-00714] Tolentino L. R., Garcia P. R. J. M., Lu V. N., Restubog S. L. D., Bordia P., Plewa C. (2014). Career adaptation: The relation of adaptability to goal orientation, proactive personality, and career optimism. Journal of Vocational Behavior.

[B46-behavsci-16-00714] Vignoli E. (2015). Career indecision and career exploration among older French adolescents: The specific role of general trait anxiety and future school and career anxiety. Journal of Vocational Behavior.

[B47-behavsci-16-00714] Wang D., Hou Z. J., Ni J., Tian L., Zhang X., Chi H.-Y., Zhao A. (2020). The effect of perfectionism on career adaptability and career decision-making difficulties. Journal of Career Development.

[B48-behavsci-16-00714] Wang L., Yan F. (2018). Emotion regulation strategy mediates the relationship between goal orientation and job search behavior among university seniors. Journal of Vocational Behavior.

[B49-behavsci-16-00714] Xu H., Tracey T. J. G. (2014). The role of ambiguity tolerance in career decision making. Journal of Vocational Behavior.

[B50-behavsci-16-00714] Yousefi Z., Abedi M., Baghban I., Eatemadi O., Abedi A. (2011). Personal and situational variables, and career concerns: Predicting career adaptability in young adults. The Spanish Journal of Psychology.

[B51-behavsci-16-00714] Yu H., Dai Y., Guan X., Wang W. (2020). Career adapt-abilities scale–short form (CAAS-SF): Validation across three different samples in the Chinese context. Journal of Career Assessment.

[B52-behavsci-16-00714] Zhang L., Wang X., Chen R., Fan W., Li H. (2025). Subjective social class and work volition: A longitudinal mediation model of career decision self-efficacy. The Career Development Quarterly.

[B53-behavsci-16-00714] Zhou M., Xu Y. (2014). Chinese university students’ achievement goals and career decision-making. International conference on education and social sciences (INTCESS14).

[B54-behavsci-16-00714] Zhu J., Hou Z., Zhang H., Wang D., Jia Y., Flores L. Y., Chen S. (2023). To Be Successful and/or Comfortable? Parental career expectations and Chinese undergraduates’ career indecisiveness across gender. Journal of Career Development.

